# A New Breast Cancer Model

**DOI:** 10.1371/journal.pbio.0020059

**Published:** 2004-02-17

Thanks to the tools of molecular biology, our understanding of the 100-plus diseases known collectively as cancer has increased dramatically over the past decades. While each of these cancers exhibits unique characteristics reflecting the particular cell or tissue it springs from, the disease follows a similar arc in nearly all its forms. Cancer is a multistep disease that begins when genetic damage—initiated by a multitude of agents—unleashes a single cell from the normal constraints on cellular proliferation. This single transformed cell generates a colony of similarly abnormal progeny that can take decades to develop into malignancies.

While events that stimulate uncontrolled cell division can promote cancer, mutations in tumor suppressor genes figure prominently in tumor progression. Disruptions in the pRb (retinoblastoma 1) tumor suppressor, for example, are often seen early in cancer development, sensitizing cells to tumorigenesis. pRb, along with other “pocket proteins”—so-called because they share an amino acid domain called the Rb pocket—regulate cell cycle progression, apoptosis (programmed cell death), and cellular differentiation. Some tumor suppressors, such as p53, can trigger apoptosis, ultimately sacrificing cells that have sustained DNA damage or other types of cellular stress.

Mutations in both the pRb and p53 tumor suppressor pathways are commonly seen in human cancers, though their interactions appear to vary depending on the tissue. In mouse brain epithelial cells, for example, loss of p53 function coupled with loss of pRb results in reduced apoptosis and increased tumor growth, while p53 loss in mouse brain astrocytes (cells that support neurons) does not affect tumor growth. Building on this work, Terry Van Dyke and colleagues report that loss of the pRb tumor suppressor in mammary tissue has the same effect—predisposition to tumor formation—seen in these other cell types. Despite the different environment inherent in each cell type, the initial events following loss of the pRb pathway were the same: increased proliferation and apoptosis, followed by tumorigenesis. But, surprisingly, pRb and p53 interactions varied in different cell types.

Like most cancers, mammary gland cancer has a long latency period, prompting the researchers to ask what events engineer tumor progression. To investigate the relative contribution of pRb and p53 in tumorigenesis, the researchers generated a novel mouse model with a dysfunctional pRb pathway and various levels of p53 function in several cell types. This is a significant achievement in itself, as many agents that inactivate the pRb pathway also disrupt the p53 pathway. pRb inactivation, they show, causes abnormalities in mammary cell proliferation, apoptosis, and tissue morphology. In these mammary-specific pRb-deficient mice, p53 was responsible for most of the apoptotic response—decreased levels of p53 resulted in reduced apoptosis and accelerated tumorigenesis, but had no effect on proliferation. Interestingly, in other mouse models where aberrant proliferation is caused by disabling other pathways, loss of p53 was associated with increased proliferation—rather than reduced apoptosis—and early tumor formation. And while p53 is the main effector of apoptosis in brain and mammary epithelial cells, this is not the case in all tissues: in astrocytes, for example, the tumor suppressor Pten regulates apoptosis in response to pRb inactivation. Together these results indicate that specific cellular responses to a cancer-causing stimulus vary depending on the nature of the initial genetic injury and the cell type and that pRb and p53 interact in different ways in different tissues. And p53, it appears, contributes to tumor suppression—and thus progression—through multiple mechanisms.

By creating a mouse model that disentangles the pRb and p53 pathways, Van Dyke and colleagues have added a valuable resource for studying breast cancer. This model, they propose, will facilitate further investigations into the relative contributions of these overlapping pathways to cancer progression. What's more, the model offers a vehicle for examining how pRb interacts with other breast cancer mutations, like the inherited mutations in the human *BRCA1* and *BRCA2* genes, to shed light on the complex series of events that ultimately cause breast cancer.

**Figure:**
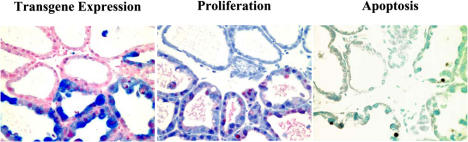
Transgene expression is associated with increased cell proliferation and cell death (apoptosis)

